# Supplementary material for: Crystallographic and spectroscopic assignment of the proton transfer pathway in [FeFe]-hydrogenases
Source: Nat Commun. 2018 Nov 9;9:4726. doi: 10.1038/s41467-018-07140-x (PMC6226526; doi:10.1038/s41467-018-07140-x)
Supplement: Supplementary file 1 — Supplementary Information [file 41467_2018_7140_MOESM1_ESM.pdf]

Supplementary Information

Crystallographic and spectroscopic assignment of the proton transfer pathway in [FeFe]-hydrogenases

J. Duan et al.

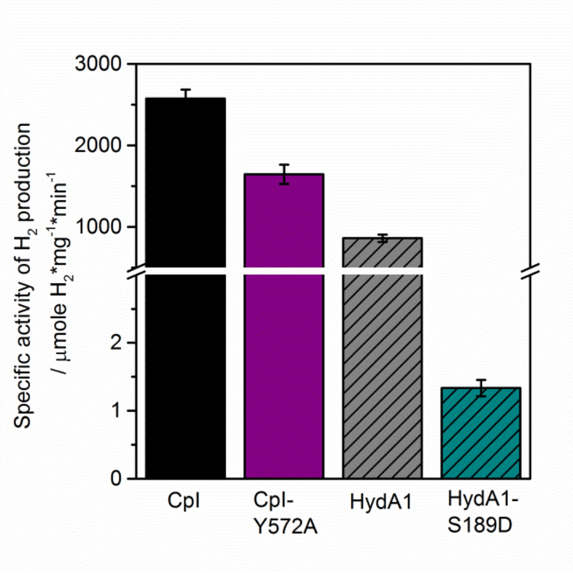

Supplementary Figure 1│ Specific H_2-production activities of CpI and HydA1 wild type enzymes and selected SDM-variants. Absolute values for MV-specific *in vitro* H_2-production activities are compared. The assay solution contained 400 ng enzyme in 100 mM potassium phosphate (pH 6.8) buffer, supplemented with 10 mM methyl viologen as electron mediator and 100 mM sodium dithiolate as sacrificial electron donor. The samples were prepared in a glove box under anaerobic conditions. After purging the samples with Ar for 5 min, the sealed suba vessels were incubated for 20 min at 37°C in a shaking water bath before 400 µl head-space was analyzed via gas-chromatography. All activities represent mean values (± standard deviation) from at least three independent measurements.

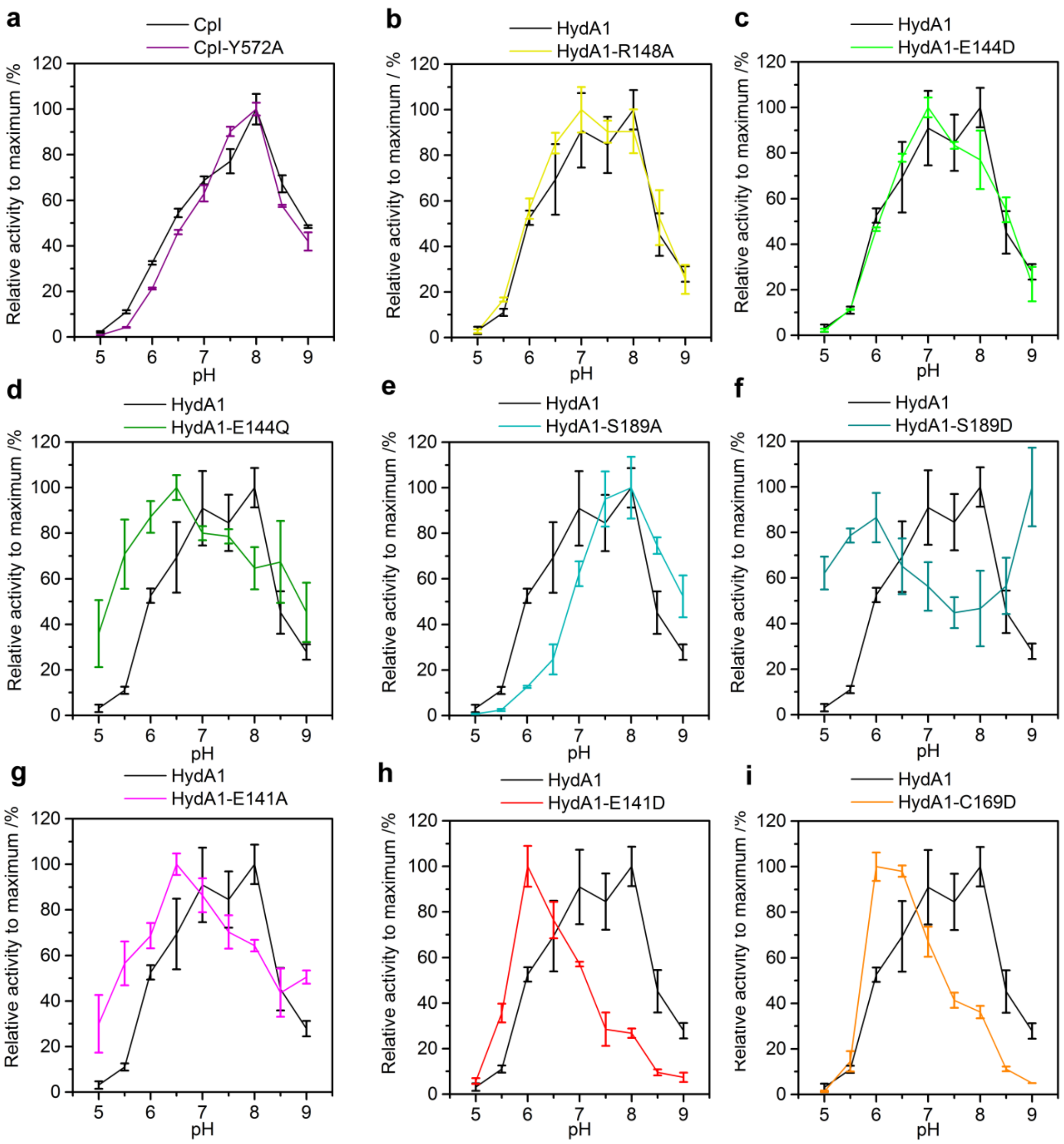

Supplementary Figure 2│ PH-activity profiles for H_2-evolution activity of selected CpI and HydA1 variants compared to wild type proteins. (a): Variant CpI-Y572A targeting a position outside the PT-pathway reproduces the pH-activity profile of wild type CpI. (**b-i**)**:** PH-activity profiles for H_2-evolution activity of HydA1 variants targeting the PT-pathway exhibit similar trends as corresponding PT-pathway variants of CpI (**see Fig. 2**). Relative values correspond to % of maximum activity obtained throughout the entire pH gradient. Error bars represent standard deviations resulting from at least three independent measurements. Color scheme corresponds to the one used in **Fig. 2.**

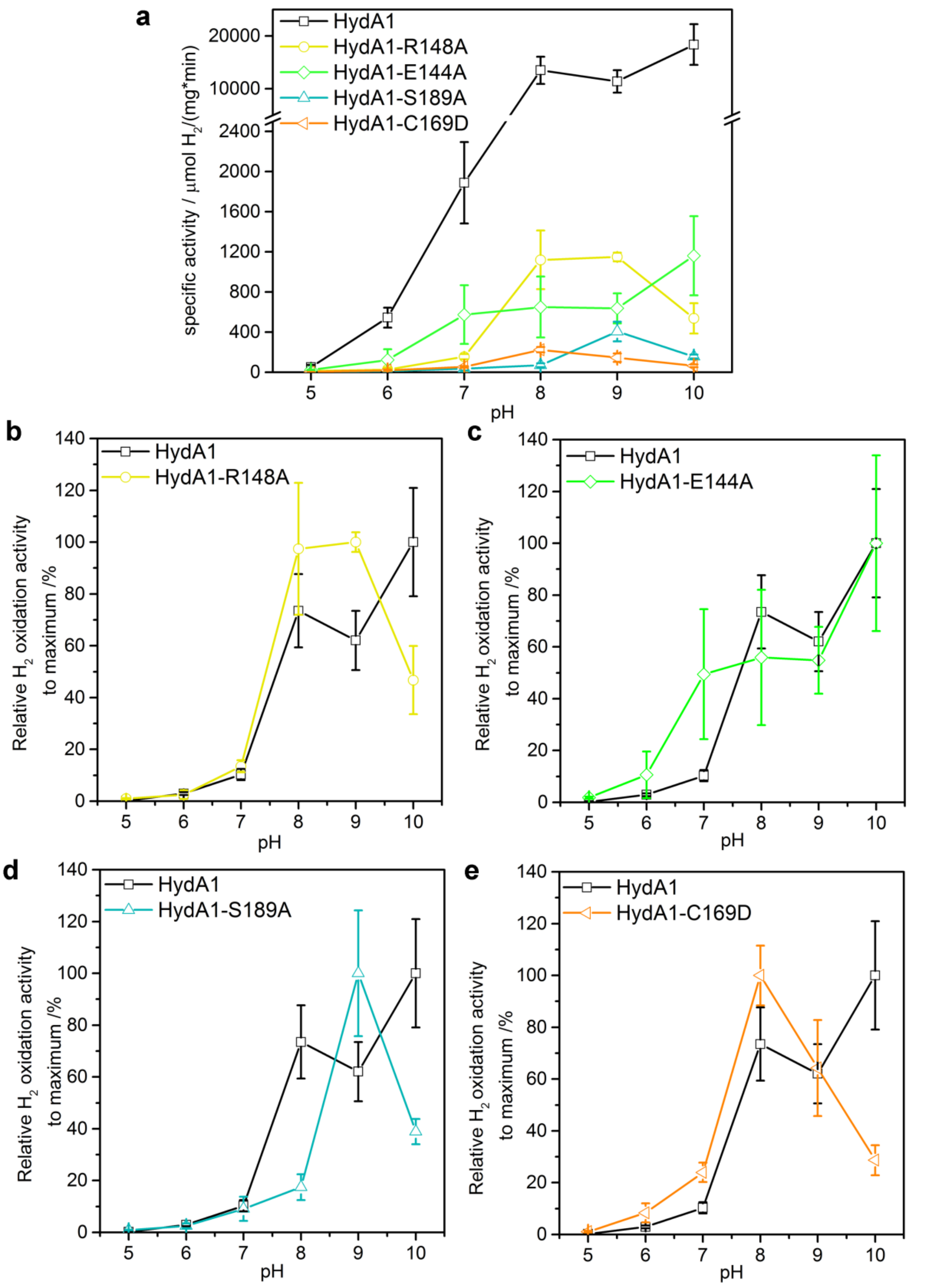

Supplementary Figure 3 │PH-activity profiles of absolute (a) and relative (b-e) H_2-oxidation activity for selected SDM-variants targeting the PT-pathway. Maximum activities of HydA1-WT, -R148A, E144A, -S189A and –C169D were obtained as 18375, 1150, 1162, 405 and 225 µmol H_2 mg^{-1} min^{-1} at pH 10, 9, 10, 9 and 8, respectively. H_2-oxidation activity of 0.5 ng -1000 ng of purified enzyme was assayed using 10 mM benzyl-viologen (BV) as electron acceptor under 1 bar of H_2. H_2-turnover was indirectly calculated from the rate of H_2-dependent BV-reduction which was spectrophotometrically determined by monitoring the absorbance at 600 nm (ε_{600}:10 $mM^{-1}cm^{-1}$)[1,2]. Relative values correspond to % of maximum activity obtained throughout the entire pH gradient. Error bars represent standard deviations resulting from at least three independent measurements.

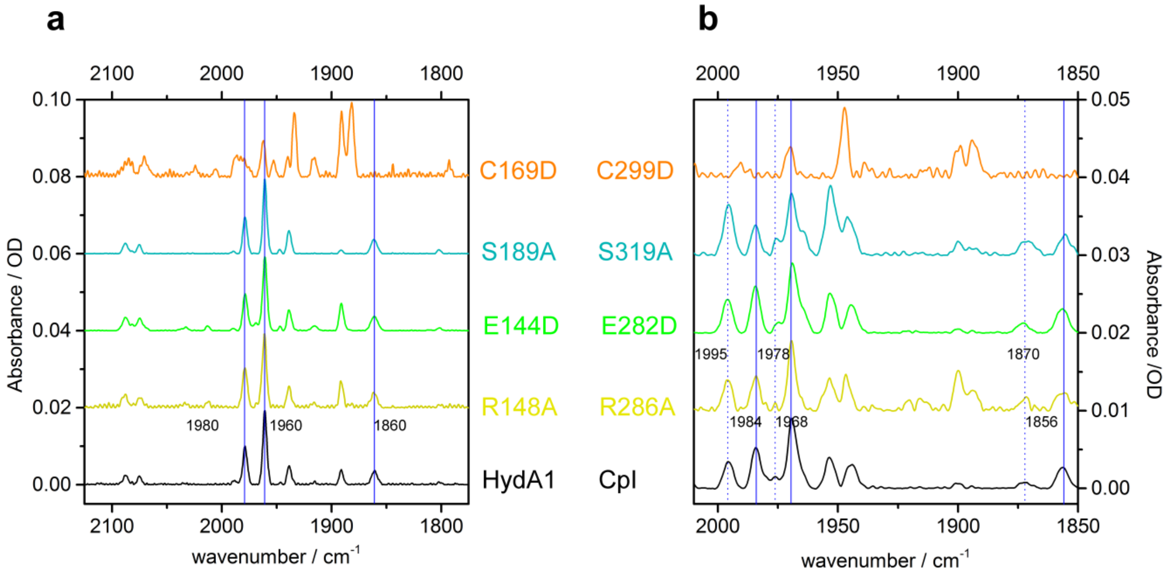

Supplementary Figure 4 | FTIR spectra of wild type enzymes and selected PT-pathway variants of HydA1 (a) and CpI (b) recorded under H_2 atmosphere at pH4. Solid and dashed blue lines indicate wavenumbers of vibrational signals from CO-ligands in H_{hyd} and $H_{hyd}H$, respectively[3].

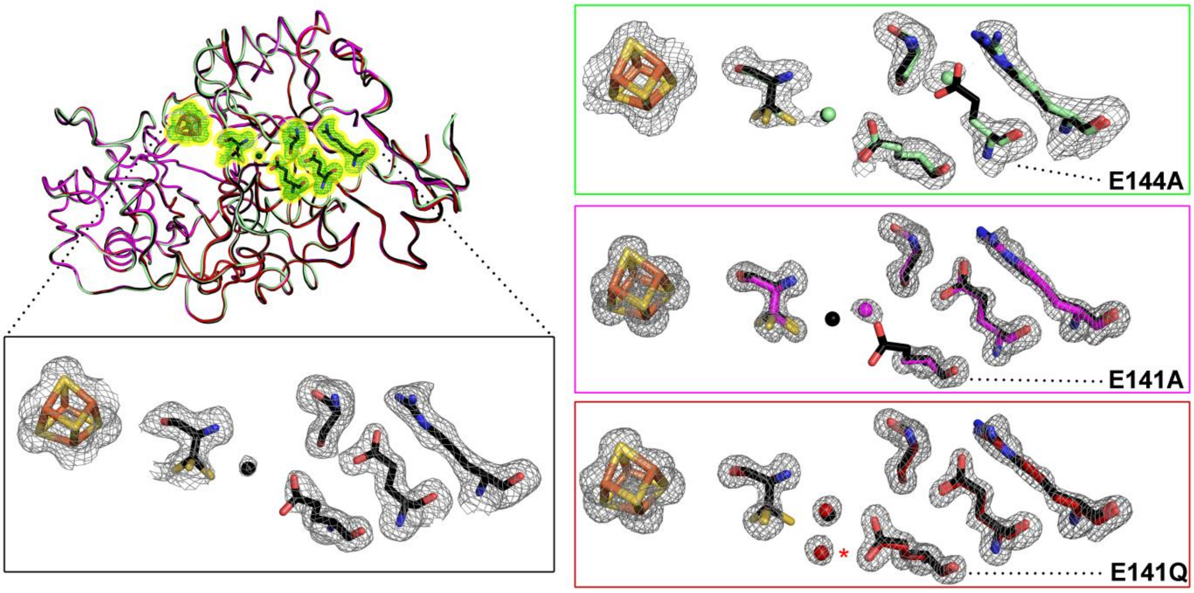

Supplementary Figure 5│ Structural features of variants targeting the putative PT-pathway in HydA1. Variant structures are superimposed as cartoon-loop models together with the wild type structure (3LX4[4] chain A). No unspecific differences are observed. For each variant an enlargement of its electron density map in the putative PT-pathway and the corresponding sticks model have been aligned with the structure of wild type protein (H_2O molecules and carbon atoms are colored in black). Simulated annealing omitting maps (F_o-F_c) were contoured at 3σ (E141A and E141Q) and 2σ (3LX4[4] and E144A). The color codes correspond to **Fig. 4**.

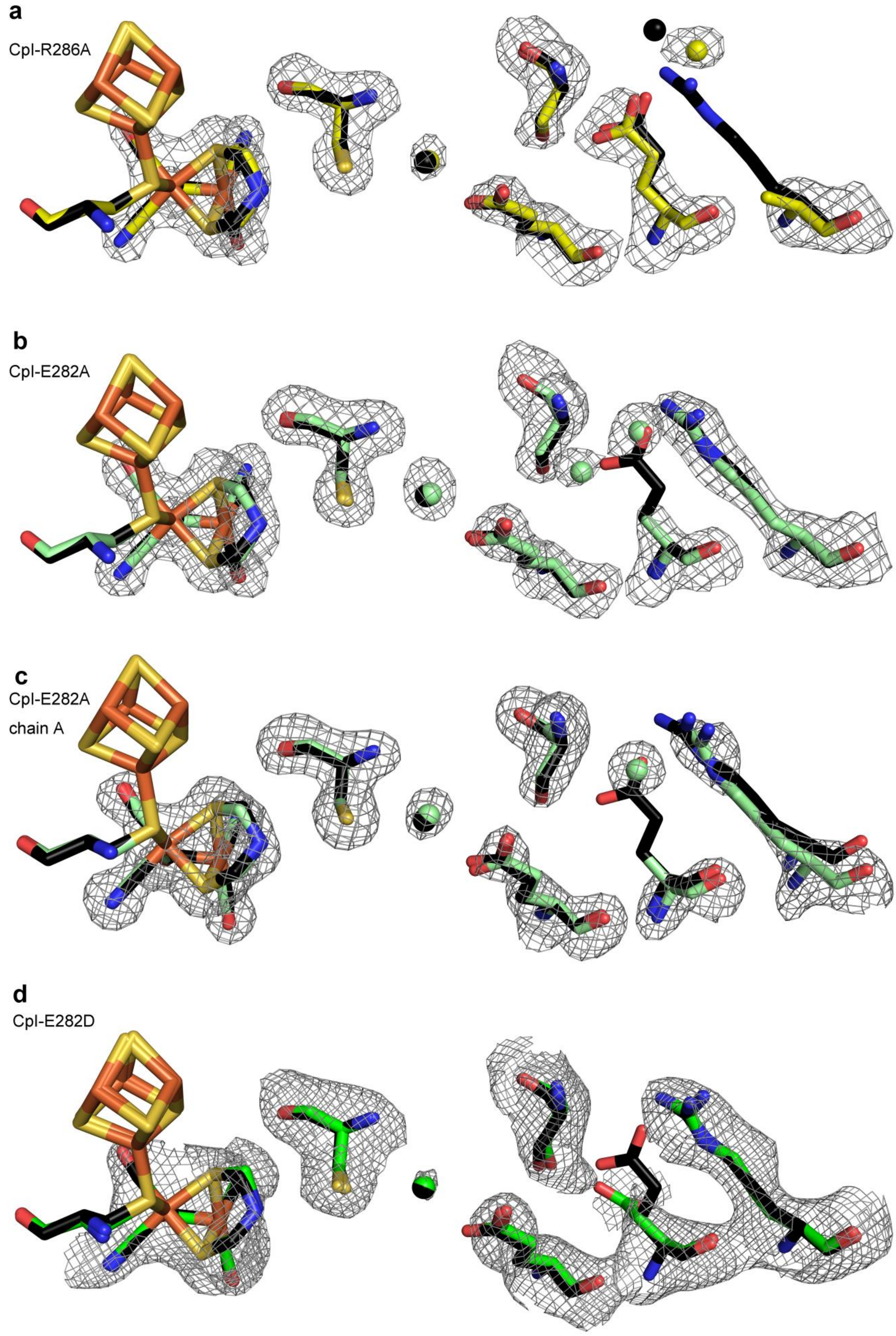
a
CpI-R286A
b
CpI-E282A
c
CpI-E282A
chain A
d
CpI-E282D

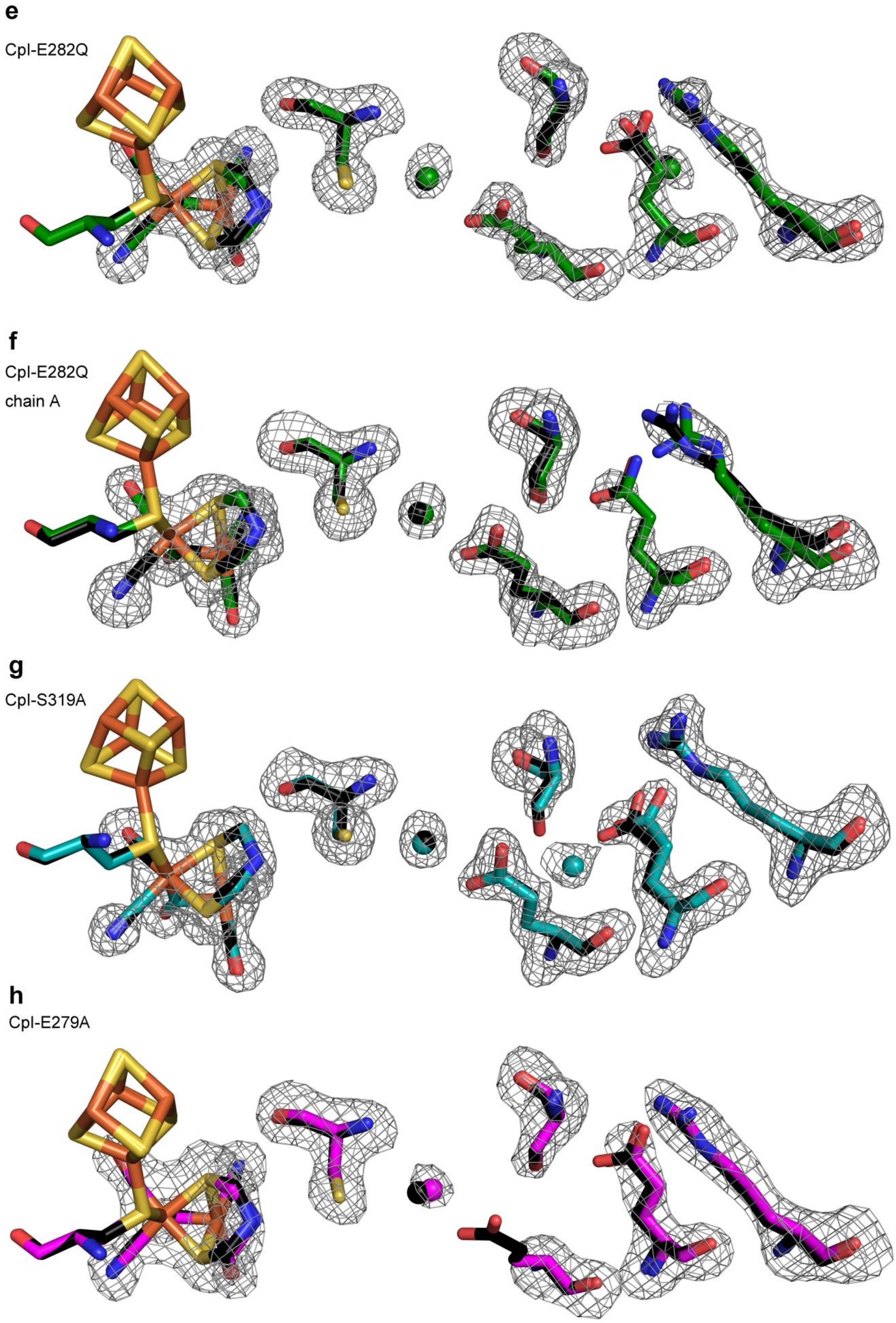

e
Cpl-E282Q
f
Cpl-E282Q
chain A
g
Cpl-S319A
h
Cpl-E279A

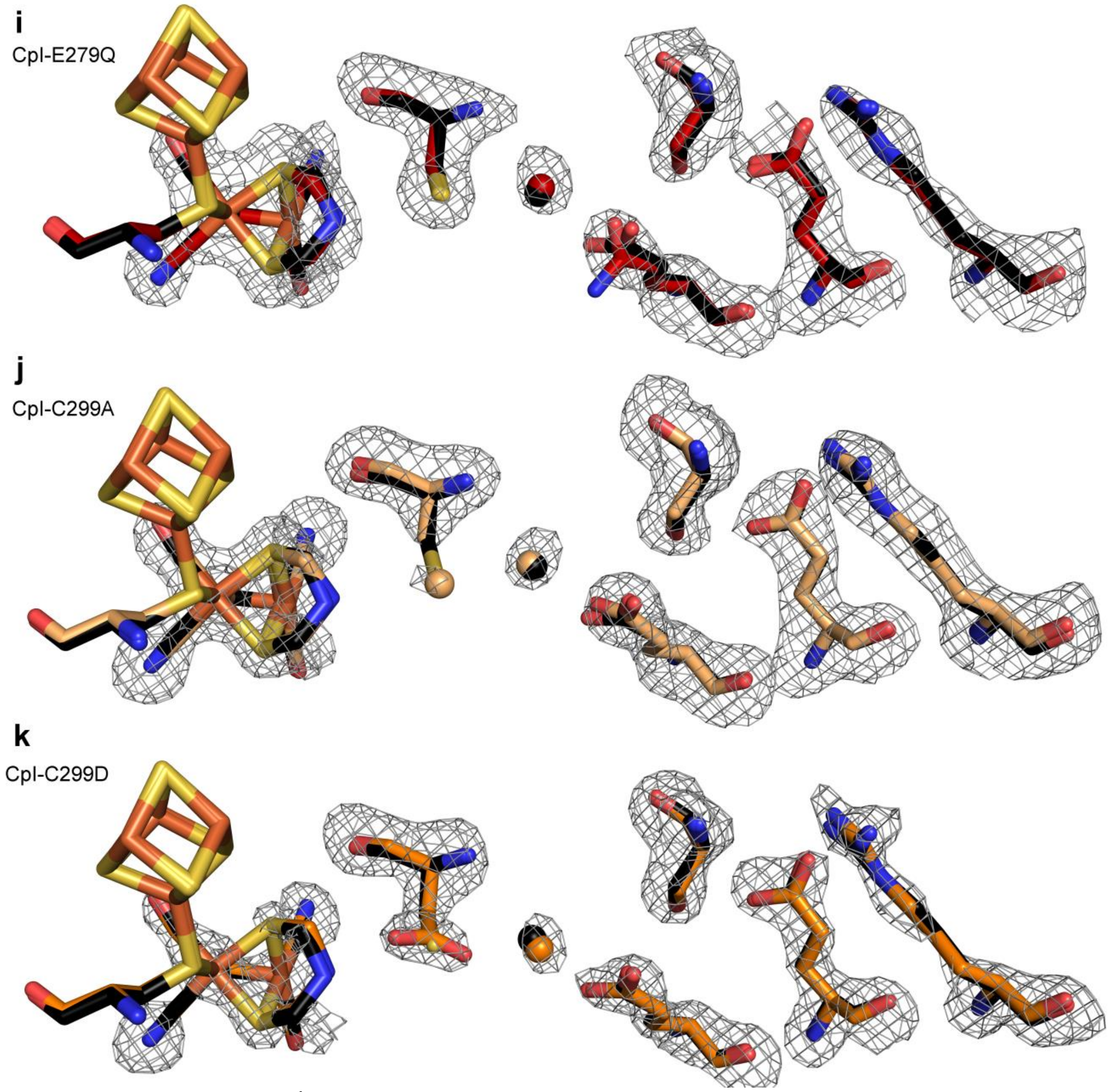

Supplementary Figure 6 │Electron density maps and structure models of the PT-pathway derived from crystal-structures of selected CpI-variants. Variants structures (**a:** R286A chain B; **b-c:** E282A chain B and A; **d:** E282D chain B; **e-f:** E282Q chain B and A; **g:** S319A chain B; **h:** E279A chain B; **i:** E279Q chain B; **j:** C299A chain B; **k:** C299D chain B) are superposed with the stick structure model of the native pathway in wild type CpI (4XDC[5]). The color code corresponds to the one used in **Fig. 3**. Simulated annealing electron density omit-maps (F_o-F_c) are presented together with the corresponding stick structure models of amino acids and H_2O molecules in the PT-pathway. The omit-maps were contoured at 3 σ except for E282D (**d**, at 1.9 σ) and E282Q chain A (**f**, at 2σ).

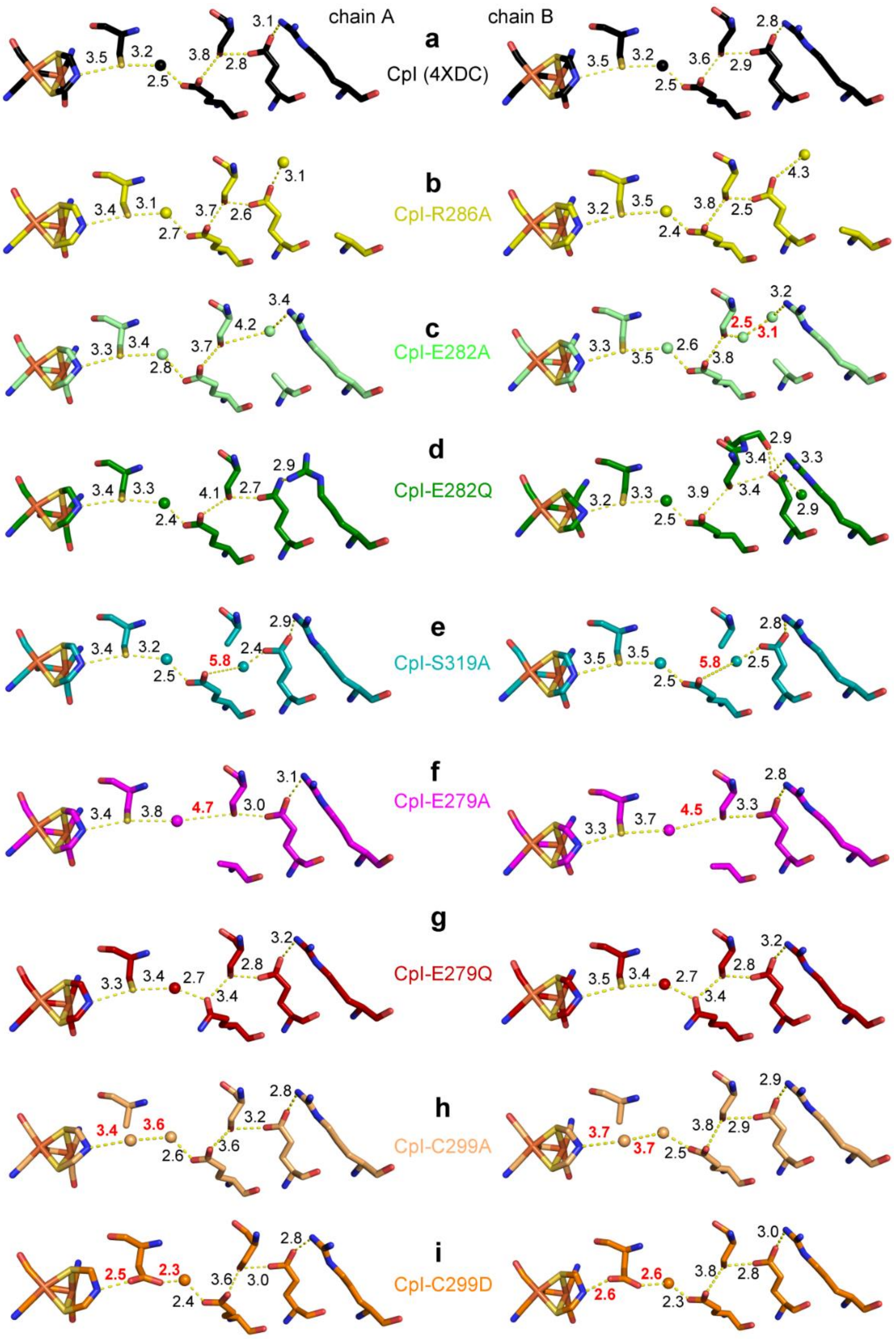

Supplementary Figure 7 │ Distances (Å) between neighboring H-bond partners in the PT-pathway of copies A (left) and B (right) in crystal structures of CpI WT (4XDC[5]) and SDM variants. PT-pathways of wild type CpI(**a**) and variants R286A(**b**), E282A(**c**), E282Q(**d**), S319A(**e**), E279A(**f**), E279Q(**g**), C299A(**h**) and C299D(**i**) are presented as stick structures with water molecules shown as spheres. Distance markers are presented as broken yellow lines with black distance labels. Distances labels potentially relevant for explaining the effects of the respective substitution on proton transfer and enzyme activity are shown in bold red numbers.

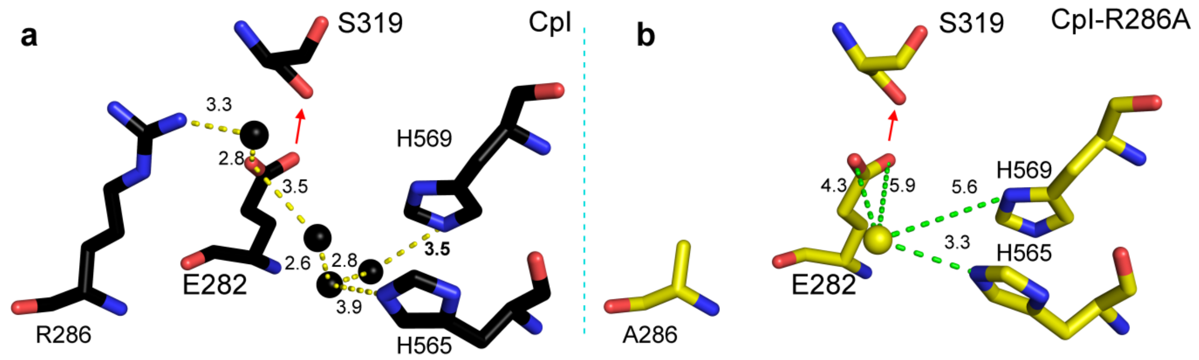

Supplementary Figure 8 │ Distances of surface water molecules at the entrance of the PT-pathway in CpI-WT (a) and variant R286A (b). Stick models of surface residues of the PT-pathway and closely located His residues (H565 and H569) are shown and distinguished by different colors of carbon atoms (black for WT (4XDC)[5] and yellow for R286A). The oxygen atoms of water molecules are shown as spheres and colored in correspondence to the carbon atoms. The color code corresponds to the one used in **Fig. 4**. Red arrow illustrates the direction of proton movement through the PT-pathway.

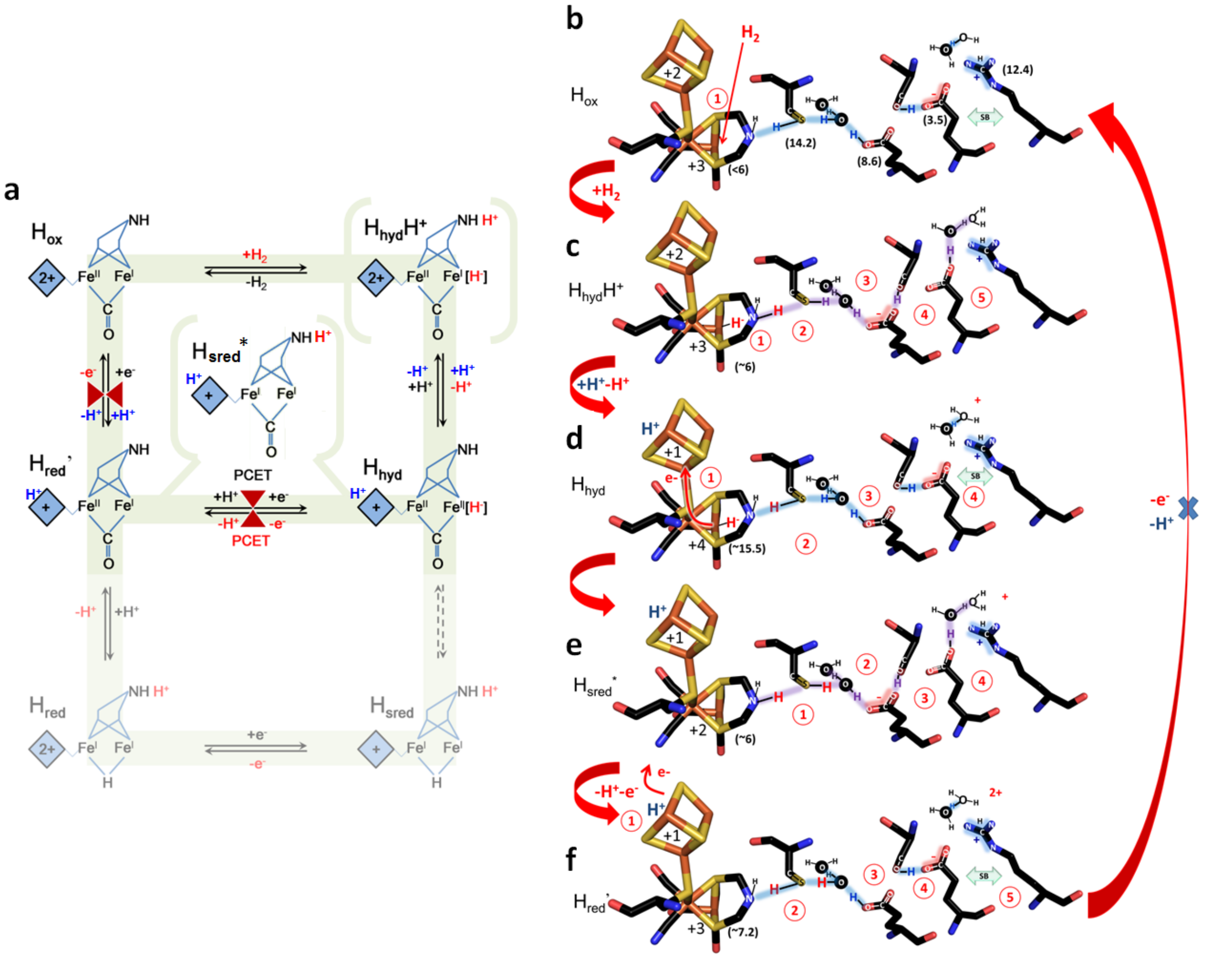

Supplementary Figure 9 | Model for the sequence of electron- and proton-transfer steps in the catalytic cycle of [FeFe]-hydrogenases. a: Proposed catalytic mechanism at the H-cluster of [FeFe]-hydrogenases, taking into account the most recent publications on the respective topic[3,6-10]. The previously discussed fast turnover cycle, involving at least three states (H_{ox}, H_{hyd} and H_{red}') is shown in the upper part. The hypothetical states $H_{hyd}H^+$ and H_{sred}^* (marked by parentheses) with a protonated adt-ligand which precede/follow H_{hyd} or H_{red}' might be regarded as transition states. Omitting H_{sred}^* the transition from H_{hyd} to H_{red}' can be viewed as a proton-coupled electron transfer step (PCET). The two bridging H^- (μ-H^-) states H_{red} and H_{sred} are not included in the fast turnover cycle but can accumulate under non-turnover conditions (half transparent lower part). The interconversion between H_{hyd} and H_{sred} is under debate (indicated by dashed arrows). **b-f:** Two H-bond patterns of the PT-pathway, differentiated by blue and purple background colors alternate in four of the five proposed catalytic states. Upon each shift in the H-bond pattern, the protons in the PT-pathway move one step further away from the $[2Fe]_H$-cluster to the surface. Only the H_{red}'$\rightarrow H_{ox}$ transition doesn't induce a change of the H-bond pattern as the $[2Fe]_H$-cluster is already deprotonated. To correlate this model with the states accumulated under the conditions of the ATR-FTIR-experiment, steps implicating electron release from the H-cluster, (especially the second one leading from f back to b) are unlikely due to the lack of external electron acceptors.

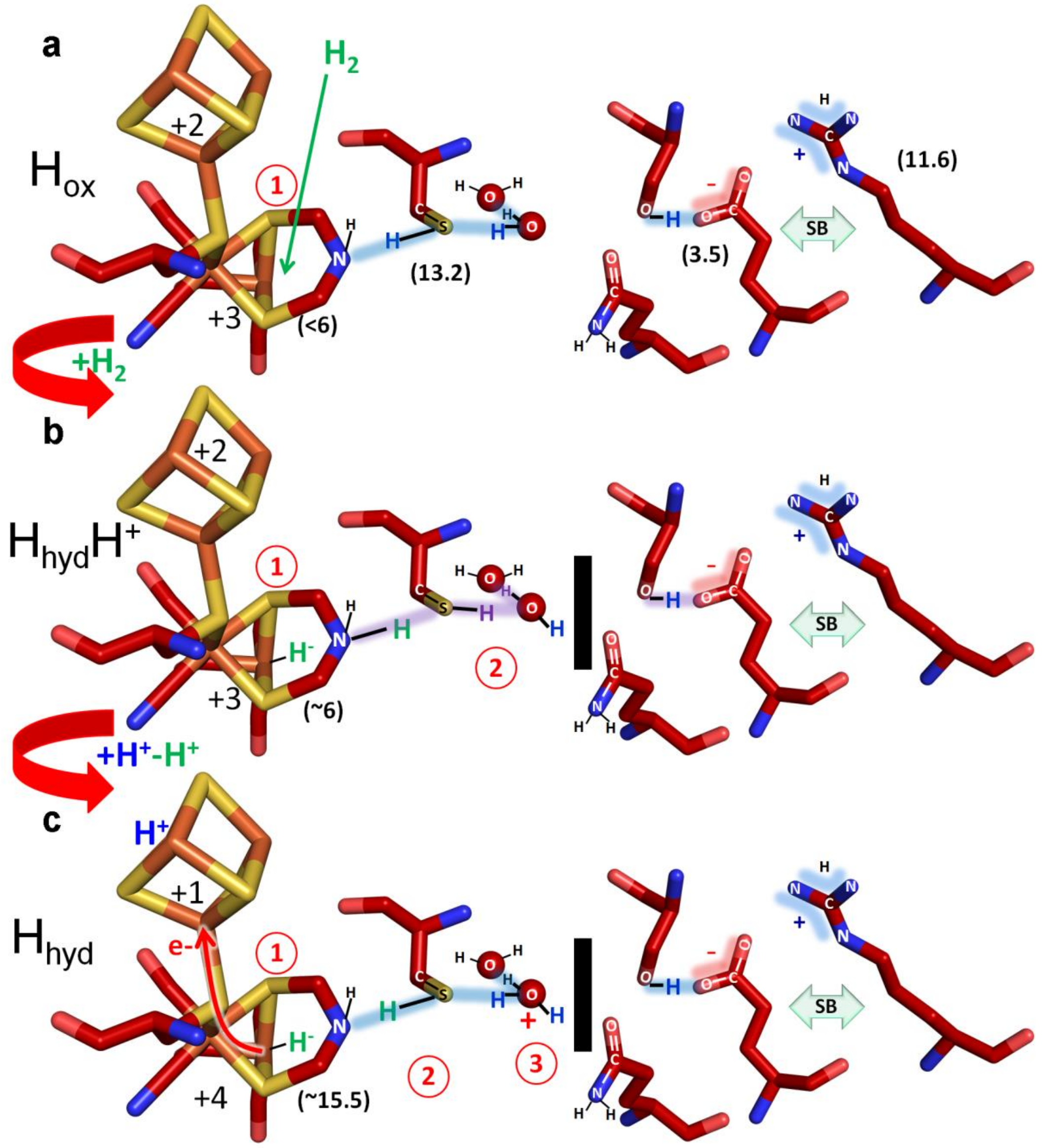

Supplementary Figure 10 │ Model of the proton transfer mechanism in variant CpI-E279Q. Depicted are stick structure models of the PT pathway in chain B of the crystal structure of variant E279Q, annotated with the presumptive H-bond patterns in the successive catalytic states H_{ox} (**a**), $H_{hyd}H^{+}$ (**b**) and H_{hyd} (**c**) after H_2-uptake. Being a consequence of the E→Q exchange at position 279, the H_2O-cluster is incapable of exchanging protons with the downstream part of the proton transfer pathway (Q279; S319; E282; R286). The pathway is interrupted and cannot be rescued by H_2O molecules, as this position is less accessible for surface water and the substituting Gln residue occupies the required space. The double arrow labeled with SB indicates a putative salt bridge contact between the protonated residue of R286 and deprotonated E282.

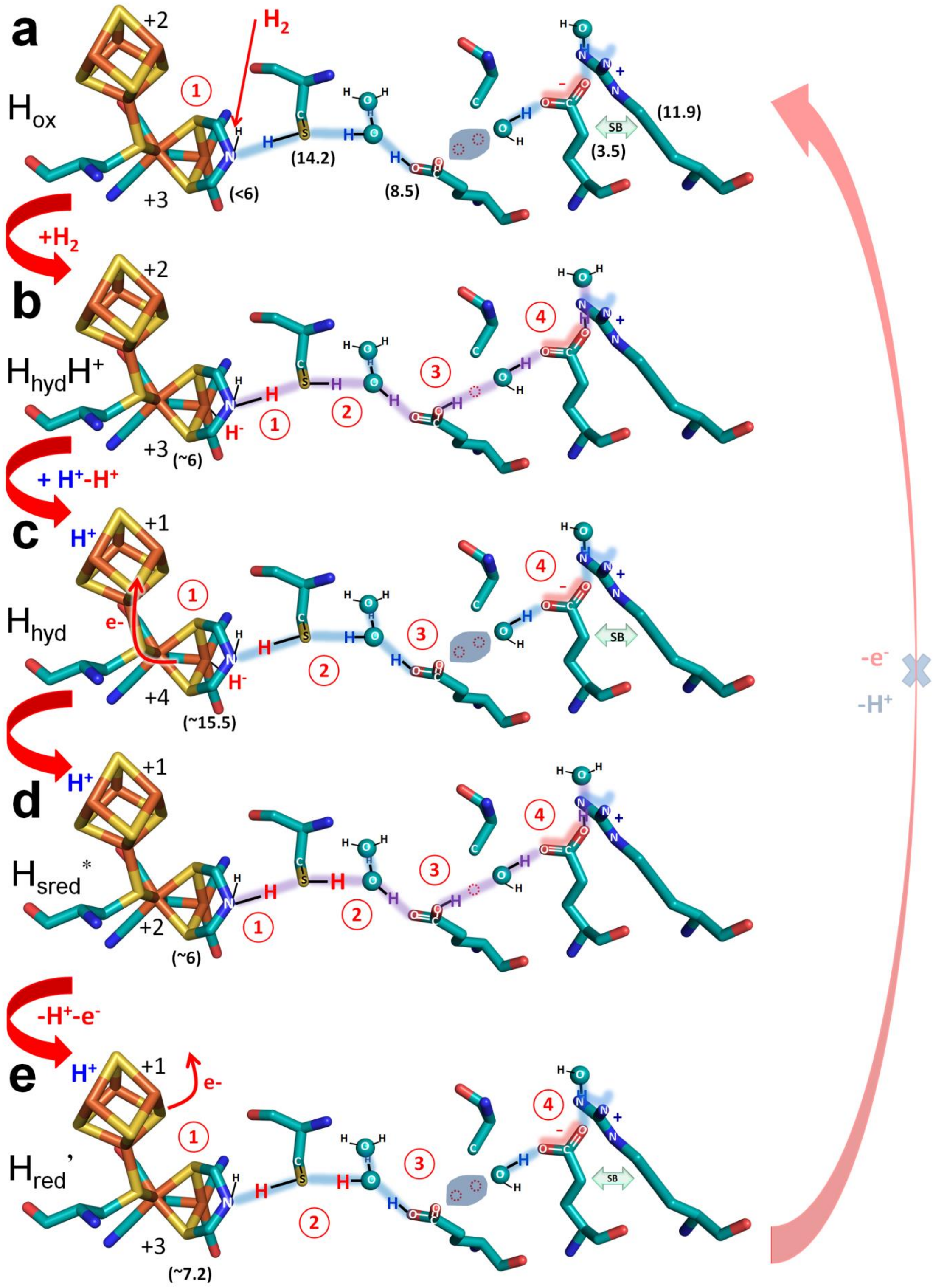

Supplementary Figure 11 │ Model of the proton transfer mechanism in variant CpI-S319A. Depicted are stick structure models of the PT pathway in chain B of the crystal structure of variant S319A, annotated with the presumptive H-bond patterns in the successive catalytic states H_{ox} (**a**), $H_{hyd}H^{+}$ (**b**), H_{hyd} (**c**), H_{sred}*(**d**) and H_{red}'(**e**) after H_2-uptake. The dash cycles indicate assumed water molecules bridging the gap between the ordered water molecule and E279. The double-arrow labeled with SB indicates a putative salt bridge contact between the protonated residue of R286 and deprotonated E282.

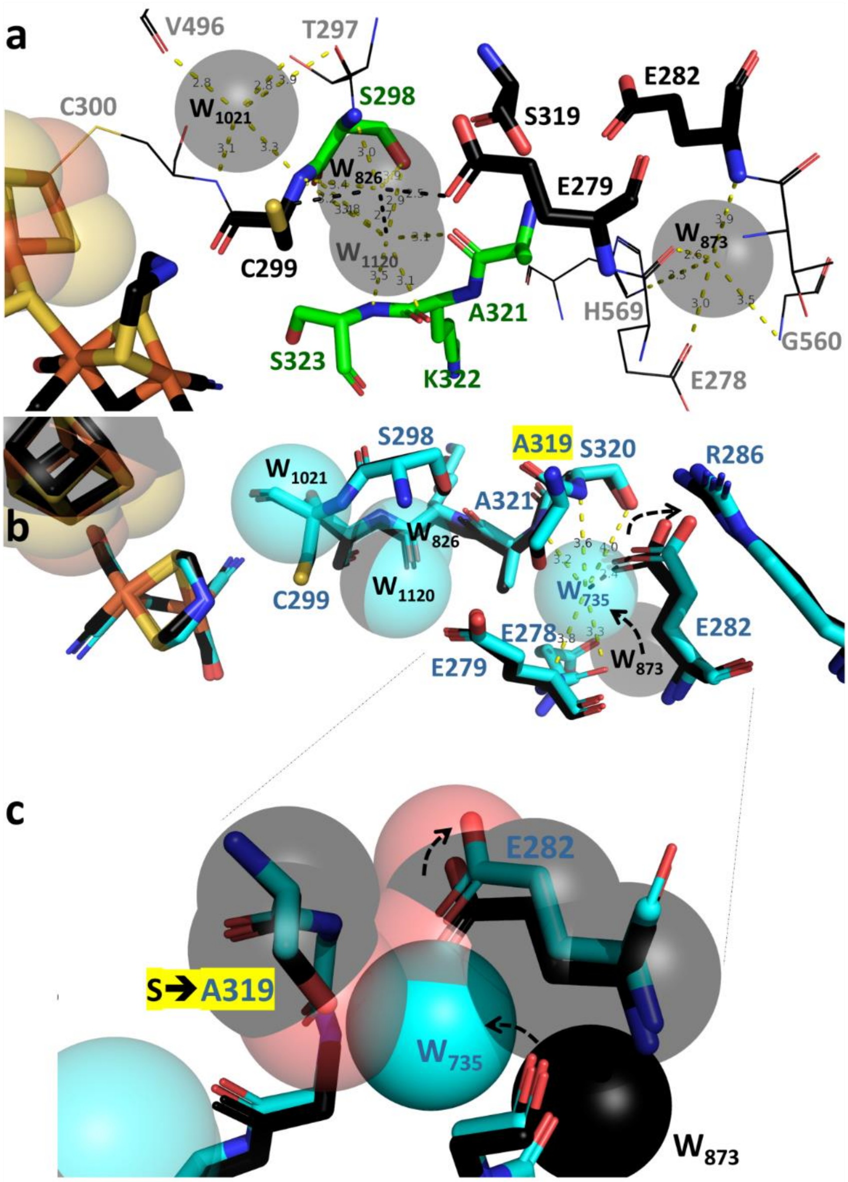

Supplementary Figure 12| H-bond networks stabilizing protein bound water molecules which participate in proton transfer in CpI wild type and S319A. Only H-bond markers for distances ≤4Å are presented. H-bond distances between water molecules or to residues of the proton transfer pathway are depicted in black. H-bond contacts of H_2O-molecules to the protein environment are presented in yellow. **a:** H-bond networks for W862, W1120 (carbon atoms of amino acids with participating main chain and side chain groups are marked in green) and W873 (carbon atoms of amino acids with participating main chain and side chain groups are marked in grey) in CpI wild type. **b:** H-bond network stabilizing W735 in S319A$_{CpI}$ shifted from its original position in wild type protein (W873) as a consequence of the amino acid exchange. Stick structures of wild type CpI (4XDC; black) and S319A$_{CpI}$ (cyan) have been superposed to compare the original and translocated position of the water molecule with the H-bond network changing from five potential contacts to main and side chain groups of E282, G560, E278 and H569 to six contacts with E282, E279, A319, A321 and S320. **c:** Enlargement of the two water positions in CpI wild type and S319A$_{CpI}$ demonstrating the steric hindrance of W873 translocation in wild type (black) due to the presence of the hydroxyl group of S319 and the conformation of E282. Its carboxy group is slightly twisted inwards as a consequence of the H-bond contact with S319.

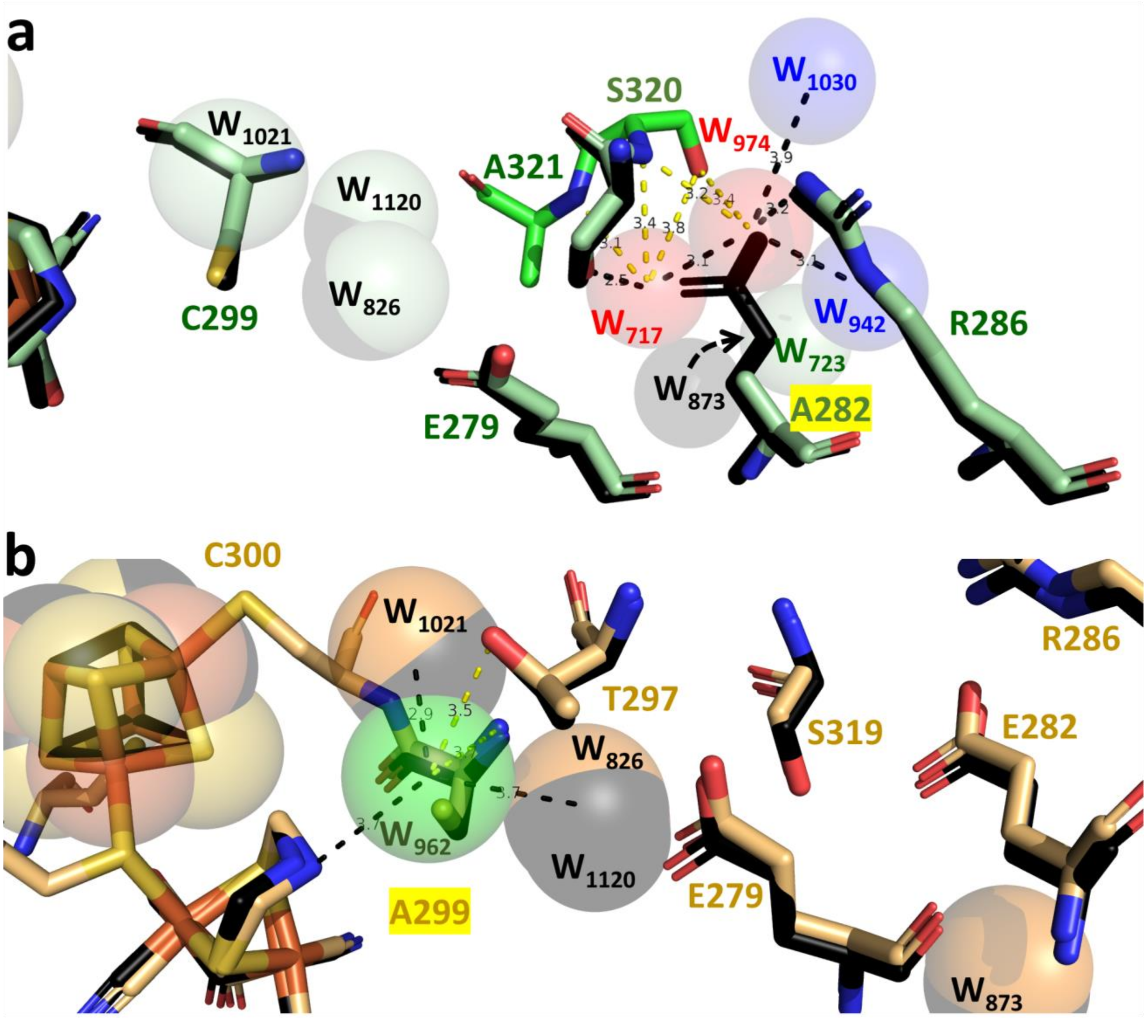

Supplementary Figure 13| H-bond networks stabilizing additional protein-bound water molecules in the proton transfer pathways of CpI variants E282A and C299A. Only H-bond markers for distances ⩽4Å are presented. Stick structures of wild type CpI (4XDC; black stick structures and water molecules presented as spheres) and E282A$_{CpI}$ (**a**) (mint colored green stick structures and water molecules, presented as spheres) or C299A$_{CpI}$ (**b**) (light orange and water molecules) have been superposed to compare the positions of the translocated water molecule in both proteins and additional H_2O molecules are depicted in red (**a**) and green (**b**), respectively. Blue spheres depict water molecules which are located at the protein surface. H-bond distances between water molecules or to residues of the proton transfer pathway are depicted in black. H-bond contacts of H_2O-molecules to the protein environment are presented in yellow. Residues and water molecules are labeled in corresponding colors, with the substitute amino acid of each variant marked by a yellow background. W717and W974 of E282A$_{CpI}$ are coordinated by H-bonds to main chain and side chain groups of S319, S320, A321 and surface water molecules W1030 and W942. The additional H_2O molecule in C299A$_{CpI}$ (W962) exhibits five potential H-bond contacts with T297, A299, W1021, W1120 and the azadithiolate ligand of the [2Fe]$_H$ cluster.

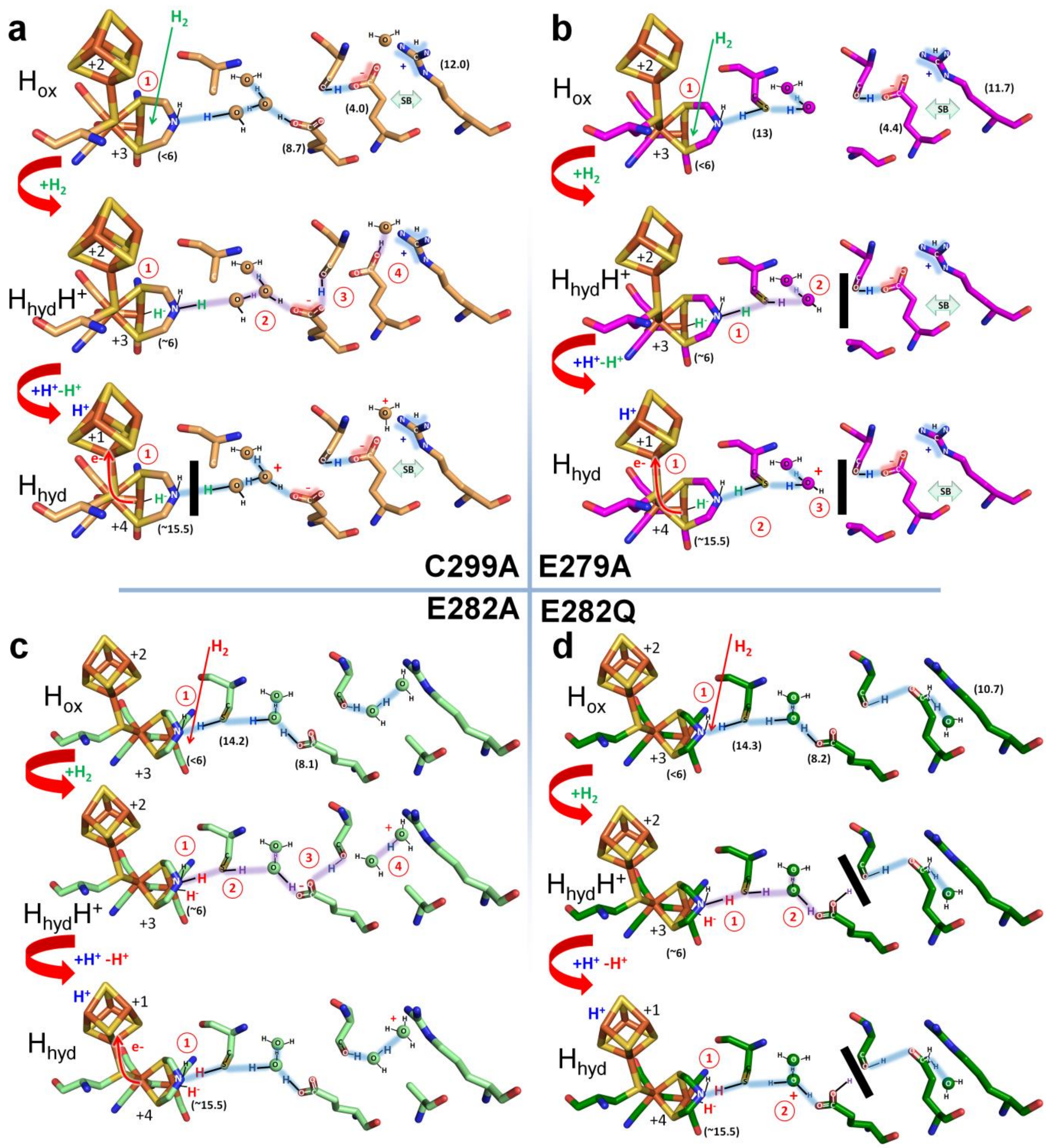

Supplementary Figure 14 | Influence of SDM on the H$^+$-transfer mechanism during H$_2$-uptake in CpI. Depicted are the presumptive effects of site directed mutagenesis on the proposed proton transfer mechanism for CpI variants C299A(**a**), E279A(**b**), E282A(**c**) and E282Q(**d**). H$_2$-binding induces a shift in the H-bond pattern (from mode 1 to mode 2) and initiates the catalytic mechanism during which the H-bond pattern repeatedly shifts between modes 1 (blue) and 2 (violet) while promoting a stepwise H$^+$-export via the PT-pathway (for details see **Supplementary Fig. 9**). Red numbers indicate locations of change which produce the respective state. The green double arrow indicates a putative salt bridge contact. Protons presented in blue close to the [4Fe]$_H$ sub-cluster originate from the recently described regulatory PT-pathway[3,11] which is independent of substrate/product transfer.

Supplementary Table1 | Summarized information on the investigated variants in this study.

Protein	H_2 production (µmol H_2 mg^{-1}min^{-1}) at pH6.8	pH optimum H_2 prodution	maximum H_2 oxidation activity (µmol H_2 mg^{-1}min^{-1}) at pH in brackets	H_{hyd} accumulation in ATR-FTIR under H_2 at either pH4 or 8	resolution of structure
**CpI-WT**	2576 ± 107	8		H_{hyd} at pH 4	1.63 Å (4XDC)
**CpI-R286A**	2285 ± 57	8		H_{hyd} at pH 4	2.22 Å
**CpI-E282A**	1470 ± 37	7		H_{hyd} at pH 8	2.05 Å
**CpI-E282D**	2099 ± 113	7.5		H_{hyd} at pH 4	2.76 Å
**CpI-E282Q**	211 ± 2.31	6		H_{hyd} at pH 8	1.96 Å
**CpI-S319A**	153 ± 11.2	7		H_{hyd} at pH 4	1.97 Å
**CpI-E279A**	1.57 ± 0.0463	6		H_{hyd} at pH 8	2.29 Å (5LA3)[10]
**CpI-E279D**	699 ± 50.5	6.5		H_{hyd} at pH 8	
**CpI-E279Q**	16.5 ± 0.0655	7.5		H_{hyd} at pH 8	2.11 Å
**CpI-C299A**	0			H_{hyd} at pH 8	2.09 Å
**CpI-C299D**	753 ± 47	6.5		no significant H_{hyd}	2.02 Å
**CpI-C299S**	1.05 ± 0.244			H_{hyd} at pH 8	
**CpI-Y572A**	1645 ± 118	8			
**HydA1-WT**	862 ± 46.5	8	18375 ± 3836 (10)	H_{hyd} at pH 4	1.97 Å (3LX4)[4]
**HydA1-R148A**	461 ± 60.5	7	1150 ± 43.3 (9)	H_{hyd} at pH 4	
**HydA1-E144A**	395 ± 80.7	6.5	1162.5 ± 394 (10)	H_{hyd} at pH 8	2.14 Å
**HydA1-E144D**	436 ± 16.7	7		H_{hyd} at pH 4	
**HydA1-E144Q**	3.72 ± 1.26	6.5		H_{hyd} at pH 8	
**HydA1-S189A**	119 ± 15.1	8	405 ± 98.4 (9)	H_{hyd} at pH 4	
**HydA1-S189D**	1.33 ± 0.12	9		H_{hyd} at pH 8	
**HydA1-E141A**	1.00 ± 0.173	6.5		H_{hyd} at pH 8	1.45 Å
**HydA1-E141D**	81.77 ± 12.7	6		H_{hyd} at pH 8	
**HydA1-E141Q**	1.20 ± 0.346			H_{hyd} at pH 8	1.61 Å
**HydA1-C169A**	0			H_{hyd} at pH 8	
**HydA1-C169D**	582 ± 236	6	225 ± 26.0 (9)	no significant H_{hyd}	
**HydA1-C169S**	0.92 ± 0.662			H_{hyd} at pH 8	

Supplementary Table 2 | Calculated R.M.S.D. (root mean square deviations, Å) of all Cα atoms when superposing variants with native CpI (equivalent chain) (a) and HydA1 (b)

a CpI

	4XDC (A)	4XDC (B)
**CpI-R286A**	0.53	0.75
**CpI-E282A**	0.56	0.75
**CpI-E282D**	0.61	0.63
**CpI-E282Q**	0.66	0.56
**CpI-S319A**	0.58	0.62
**CpI-E279Q**	0.36	0.38
**CpI-C299A**	0.63	0.48
**CpI-C299D**	0.6	0.44

b HydA1

	3LX4 (A)	3LX4 (B)
**apo-HydA1-E144A**	0.27	0.27
**apo-HydA1-E141A**	0.33	0.31
**apo-HydA1-E141Q**	0.25	0.24

Supplementary Table 3 | Crystallization conditions of the CpI and HydA1 variants and used beamlines for X-ray diffraction.

	Entry	Buffer & pH	Precipitant	Additives	Salt	Beamline
**CpI-R286A**	6GM3	0.1 M Mes[a] pH 6	23 % PEG4000[b]	17% Glycerol	0.4 M $MgCl_2$	SLS PXII
**CpI-E282A**	6GM1	0.1 M Mes pH 6	21 % PEG4000	19 % Glycerol	0.4 M $MgCl_2$	SLS PXII
**CpI-E282D**	6GM2	0.1 M Mes pH 6	21 % PEG4000	19 % Glycerol	0.4 M $MgCl_2$	SLS PXII
**CpI-E282Q**	6GM8	0.1 M Mes pH 6	20 % PEG4000	20 % Glycerol	0.4 M $MgCl_2$	SLS PXII
**CpI-S319A**	6GM4	0.1 M Mes pH 6	21 % PEG4000	19 % Glycerol	0.4 M $MgCl_2$	SLS PXII
**CpI-E279Q**	6GM0	0.1 M Mes pH 6	20 % PEG4000	20 % Glycerol	0.4 M $MgCl_2$	SLS PXII
**CpI-C299A**	6GLY	0.1 M Mes pH 6	21 % PEG4000	19 % Glycerol	0.4 M $MgCl_2$	ESRF ID23-1
**CpI-C299D**	6GLZ	0.1 M Mes pH 6	21 % PEG4000	19 % Glycerol	0.4 M $MgCl_2$	ESRF BM30A
**apo-HydA1-E144A**	6GM7	0.1 M Mes pH 6	20 % PEG4000	/	0.4 M NaCl	ESRF ID23-1
**apo-HydA1-E141A**	6GM5	0.1 M Mes pH 6	11 % PEG4000	/	0.5M NaCl	ESRF ID23-1
**apo-HydA1-E141Q**	6GM6	0.1 M Mes pH 6	15 % PEG4000	/	0.6 M NaCl	SLS PXII

a "Mes" stands for 2-(N-morpholino) ethanesulfonic acid.

b "PEG4000" stands for polyethylene glycol of molecular weight c.a. 4000

Supplementary Table 4 | Data collection and refinement statistics of crystal structures (I)

	CpI-R286A	CpI-E282A	CpI-E282D	CpI-E282Q	CpI-S319A	CpI-E279Q
**Data collection**						
Space group	P 1 2_1 1	P 1 2_1 1	P 1 2_1 1	P 1 2_1 1	P 1 2_1 1	P 1 2_1 1
**Cell dimensions**						
a, b, c (Å)	89.81, 72.89, 103.14	89.99, 72.96, 102.93	89.87, 72.82, 102.91	89.65, 72.34, 103.15	90.07, 73.33, 103.25	90.82, 73.04, 103.28
α, β, γ (°)	90.00, 96.11, 90.00	90.00, 96.32, 90.00	90.00, 96.86, 90.00	90.00, 96.73, 90.00	90.00, 96.46, 90.00	90.00, 98.44, 90.00
Resolution (Å)	48.46-2.22 (2.28-2.22)*	48.09-2.05 (2.10-2.05)	47.88-2.76 (2.83-2.76)	47.769-1.96 (2.01-1.96)	48.206-1.97 (2.02-1.97)	47.72-2.11 (2.16-2.11)
R_{merge}	0.1234 (0.672)	0.1077 (0.6263)	0.1989 (0.7434)	0.1579 (0.8868)	0.134 (0.8412)	0.1158 (1.095)
I / σ(I)	9.73 (2.07)	12.62 (2.75)	8.03 (2.04)	7.93 (2.00)	10.98 (2.01)	12.76 (2.00)
Completeness (%)	98.5 (98.1)	99.2 (98.5)	99.9 (100.0)	98.5 (97.5)	99.9 (100.0)	98.8 (97.9)
Redundancy	4.5 (4.3)	5.6 (5.7)	4.9 (4.8)	6.8 (6.5)	6.7 (6.3)	7.0 (7.2)
CC1/2	0.995 (0.751)	0.998 (0.854)	0.984 (0.723)	0.993 (0.731)	0.997 (0.726)	0.998 (0.822)
**Refinement**						
Resolution (Å)	48.46-2.22	48.09-2.05	47.88-2.76	47.769-1.96	48.206-1.97	47.72-2.11
No. reflections	60,779	82,662	34,211	92,827	94,650	76,250
R_{work} / R_{free}	0.1801 / 0.2267	0.1651 / 0.2059	0.2206 / 0.2757	0.1763 / 0.2211	0.1643 / 0.2015	0.1855 / 0.2282
**No. atoms**						
Protein	8909	8927	8934	8961	8913	8970
Ligand / ion	106 / 2	106 / 3	106 / 1	106 / 3	106 / 4	106 / 2
Water	676	829	76	868	895	594
**B-factors**						
Protein	37.80	33.30	34.70	31.30	30.60	43.00
Ligand	25.40	21.60	25.40	20.40	19.60	30.90
Water	43.40	41.00	26.60	41.60	40.30	44.30
**R.m.s deviations**						
Bond lengths (Å)	0.002	0.003	0.004	0.011	0.008	0.003
Bond angles (°)	0.610	0.684	0.779	1.125	0.944	0.582

*Numbers in brackets indicate values in the highest resolution shell

Supplementary Table 4 | Data collection and refinement statistics of crystal structures (II)

	CpI-C299A	CpI-C299D	apo-HydA1-E144A	apo-HydA1-E141A	apo-HydA1-E141Q
**Data collection**					
Space group	P 1 2_1 1	P 1 2_1 1	P 3_2 2 1	P 3_2 2 1	P 3_2 2 1
**Cell dimensions**					
a, b, c (Å)	89.74, 72.17, 103.05	89.80, 72.60, 102.99	70.91, 70.91, 154.80	70.62 70.62 154.91	70.79 70.79 155.03
α, β, γ (°)	90.00, 97.40, 90.00	90.00, 97.16, 90.00	90.00, 90.00, 120.00	90.00, 90.00, 120.00	90.00, 90.00, 120.00
Resolution (Å)	47.55-2.09 (2.14-2.09)	47.74-2.02 (2.07-2.02)	48.11-2.14 (2.20-2.14)	48.00-1.45 (1.49-1.45)	48.09-1.61 (1.65-1.61)
R_{merge}	0.1215 (0.7921)	0.2304 (1.081)	0.1654 (0.8052)	0.07928 (1.041)	0.1165 (1.174)
I / σ(I)	10.19 (2.03)	8.45 (2.01)	9.70 (2.03)	22.11 (2.24)	12.42 (2.00)
Completeness (%)	94.26 (91.03)	99.9 (100.0)	99.4 (99.8)	99.8 (97.2)	100.0 (100.0)
Redundancy	6.9 (6.6)	7.2 (7.5)	6.8 (6.1)	18.4 (11.3)	9.8 (9.9)
CC1/2	0.996 (0.771)	0.991 (0.714)	0.993 (0.504)	1 (0.751)	0.998 (0.687)
**Refinement**					
Resolution (Å)	47.55-2.09	47.74-2.02	48.11-2.14	48.00-1.45	48.09-1.61
No. reflections	73,106	86,351	25,469	79,955	59,107
R_{work} / R_{free}	0.1673 0.2086	0.2017 / 0.2486	0.1897 / 0.2268	0.1653 / 0.1969	0.1639 / 0.1992
**No. atoms**					
Protein	8959	8947	3115	3105	3108
Ligand / ion	106 / 3	106 / 3	8 / 1	8 / 2	8 / 2
Water	632	1009	382	577	533
**B-factors**					
Protein	38.10	26.80	29.40	24.10	24.40
Ligand	28.90	16.80	19.30	16.60	17.70
Water	41.70	31.70	36.90	38.30	38.60
**R.m.s deviations**					
Bond lengths (Å)	0.006	0.007	0.003	0.009	0.007
Bond angles (°)	0.856	0.84	0.712	1.224	1.034

Supplementary Table 5 | Occupancy and B-factors of the H-cluster and protein cavity[a] of [2Fe]$_H$ of CpI crystal structures

	R286A	E282A	E282D	E282Q	S319A	E279Q	C299A	C299D
**B-factors**								
2Fe$_H$ cavity (A/B)[b]	25.96 / 23.68	21.72 / 18.89	25.74 / 22.56	21.07 / 17.61	19.38 / 17.09	30.58 / 30.47	29.99 / 24.93	17.22 / 14.69
2Fe$_{H\ (A/B)}$	26.29 / 23.62	22.62 / 20.09	26.72 / 23.46	20.42 / 18.01	19.53 / 16.00	29.74 / 27.75	32.47 / 24.66	16.91 / 13.03
[4Fe4S]$_H$ (A/B)	26.26 / 21.67	19.02 / 17.70	20.60 / 18.60	20.06 / 15.47	18.22 / 16.47	30.72 / 29.21	28.90 / 24.64	16.53 / 14.36
**Occupancy**								
Average occupancy of 2Fe$_H$ (A/B)	1.00 / 1.00	1.00 / 1.00	1.00 / 1.00	0.84 / 0.85	1.00 / 1.00	0.97 / 0.95	0.71 / 0.69	0.64 / 0.64
Average occupancy of [4Fe4S]$_H$ (A/B)	1.00 / 1.00	1.00 / 1.00	1.00 / 1.00	1.00 / 0.99	1.00 / 1.00	0.97 / 0.96	0.90 / 0.88	0.96 / 0.95

a Protein cavity of the [2Fe]$_H$ refers to the residues described as before, namely, A230, P231, C299, S232, S323, P324, Q325, M353, K358, F417, V423, M497 and C503 of CpI.

b A/B indicates the corresponding values in two copies (chain A and B) of CpI crystal structures.

Supplementary Table 6 │ Primers of PT-pathway variants used for QuikChange PCR

	Forward 5'-3'	Reverse 5'-3'
**HydA1-R148A**	CTGCTGCATGCCCTGACCGA ACATCTGGAAGCGCATC	GATGTTCGGTCAGGGCATGCAG CAGTTCGCTGCCTTC
**HydA1-E144A**	GAAGGCAGCGCCCTGCTGCA TCGTCTGACC	GCAGCAGGGCGCTGCCTTCTTC CATGATGGTC
**HydA1-E144D**	GAAGGCAGCGATCTGCTGCA TCGTCTGACC	CAGCAGATCGCTGCCTTCTTCC ATGATGGTC
**HydA1-E144Q**	GAAGGCAGCCAGCTGCTGC ATCGTCTGACC	GCAGCAGCTGGCTGCCTTCTTC CATGATGGTC
**HydA1-S189A**	GTATGTGGCGAGCTGCAAAA GCCCGCAGATG	GCTTTTGCAGCTCGCCACATAC GGAATCAGATC
**HydA1-S189D**	GTATGTGGATAGCTGCAAAA GCCCGCAGATG	GCTTTTGCAGCTATCCACATACG GAATCAGATC
**HydA1-E141A**	CATCATGGAAGCGGGCAGCG AACTGCTGCATCGTC	GCAGTTCGCTGCCCGCTTCCAT GATGGTCAGATCC
**HydA1-E141D**	CATCATGGAAGATGGCAGCG AACTGCTGCATCGTC	GCAGTTCGCTGCCATCTTCCATG ATGGTCAGATCC
**HydA1-E141Q**	CATCATGGAACAGGGCAGCG AACTGCTGCATCGTC	GCAGTTCGCTGCCCTGTTCCATG ATGGTCAGATCC
**HydA1-C169A**	CAGCGCGTGTCCGGGCTGGA TTGC	CCGGACACGCGCTGGTAAACAT CGG
**HydA1-C169D**	GTTTACCAGCGATTGCCCGG GCTGGATTGCG	GCCCGGGCAATCGCTGGTAAAC ATCGGCAG
**HydA1-C169S**	CAGCTCATGTCCGGGCTGGA TTGC	CCGGACATGAGCTGGTAAACAT C
**CpI-R286A**	GGTTCAAGCTATCGAGAATA ATGGCCCTTTCCC	GCCATTATTCTCGATAGCTTGAA CCAGTTCGGTAG
**CpI-E282A**	ACCGCACTGGTTCAACGTAT C	ACCAGTGCGGTAGCCTCTTC
**CpI-E282D**	ACCGACCTGGTTCAACGTAT C	ACCAGGTCGGTAGCCTCTTC
**CpI-E282Q**	GGCTACCCAACTGGTTCAAC GTATC	TGAACCAGTTGGGTAGCCTCTT CC
**CpI-S319A**	GAATAATCTTGCCTCCGCTAA ATCCCCTCAAC	AGCGGAGGCAAGATTATTCAGC AGTTCAGG
**CpI-E279A**	TGGAAGCGGCTACCGAACTG GTTCAACG	CCAGTTCGGTAGCCGCTTCCATA ATGGTCATATC
**CpI-E279D**	TGGAAGACGCTACCGAACTG GTTCAACGTATC	CGGTAGCGTCTTCCATAATGGTC ATATCTG
**CpI-E279Q**	TGGAACAGGCTACCGAACTG GTTCAACGTATC	CGGTAGCCTGTTCCATAATGGTC ATATCTG
**CpI-C299A**	CCTCTGCGTGCCCAGGTTGG	CCTGGGCACGCAGAGGTAAACA

	GTACGTC	TTGGGAAAGGG
**CpI-C299D**	CCTCTGATTGCCCAGGTTGG GTACG	CTGGGCAATCAGAGGTAAACAT TGGGAAAGGG
**CpI-C299S**	ACCTCTAGCTGCCCAGGTTG GGTACG	TGGGCAGCTAGAGGTAAACATT GGGAAAGG
**CpI-Y572A**	GAAATCCTGCACTTTAAAGC GAAAAAATCAG	GGACCAGGCTGATTTTTTCGCTT TAAAG

Supplementary Table 7 | Frequency of CO ligands indicate different redox states of HydA1 and CpI

HydA1			
**State**	***p*CO**	***d*CO**	***µ*CO**
H_{hyd}	**1978**	**1960**	**1860**
$H_{ox}H$	1970	1946	1812
H_{ox}	1964	1940	1802
H_{red}	1915	1891	1961*
H_{sred}	1918	1882	1953*
H_{hyd}	1980	1962	1868
H_{ox}	1972	1948	1812
**CpI**			
**State**	***p*CO**	***d*CO**	***µ*CO**
$H_{hyd}H$	1995	1978	1870
H_{hyd}	1984	1968	1856
$H_{ox}H$	1975	1953	1809
H_{ox}	1970	1947	1800
H_{red}	1915	1900	1962*
H_{sred}	1922	1894	1958*

* terminal CO in reduced states

Note that some minimal shifts are possible among different variants.

Supplementary Table 8 │ B factors of the residues and water molecules in the PT-pathway of CpI structures

Structures	whole structure	299	Wat-826[a]	Wat-1120[a]	Wat-1021[a]	279	319	282	286	additional H_2O[b]
4XDC-A	36.2	23.10	26.49	25.45	25.62	29.87	27.49	36.83	36.48	
4XDC-B		22.33	24.57	22.23	22.74	25.92	24.98	29.93	28.87	
R286A-A	38.1	26.34	28.45	37.99	23.93	33.82	31.98	44.57	46.69	
R286A-B		23.49	25.57	21.86	30.17	30.78	28.28	32.63	35.09	
E282A-A	33.8	21.65	20.68	32.52	17.13	30.25	28.43	32.94	39.35	23.50
E282A-B		20.24	17.16	26.96	17.06	26.24	24.57	26.58	28.87	36.96; 17.99[c]
E282Q-A	32.1	19.11	20.96	25.64	18.23	27.64	27.98	39.30	44.99	
E282Q-B		16.10	19.28	20.28	16.89	23.70	19.83	26.40	24.66	
S319A-A	31.4	18.58	17.29	20.88	15.96	25.86	27.30	33.83	37.69	32.26
S319A-B		16.53	15.62	17.11	13.74	21.67	20.70	28.65	26.29	29.69
E279A-A	33.7	26.56	28.33	30.91	23.36	29.90	31.29	39.88	45.57	
E279A-B		17.29	27.86	21.94	19.42	21.74	24.20	28.66	25.23	
E279Q-A	42.9	31.14	29.45	36.92	31.17	39.16	37.97	44.27	47.70	
E279Q-B		33.99	30.49	33.21	31.04	37.70	39.30	39.13	41.53	
C299A-A	38.3	30.09	30.10	43.58	36.47	40.03	36.61	45.29	49.58	35.18
C299A-B		27.64	29.63	25.67	26.03	31.35	29.00	37.27	32.58	33.89
C299D-A	26.8	18.88	18.88	17.37	13.17	23.29	24.12	27.92	31.72	
C299D-B		17.37	16.11	15.17	13.50	17.09	17.37	23.81	24.22	

a: The numbering of water molecules corresponds to chain B of WT structure (4XDC[5]).

b: In structures of E282A, S319A and C299A, additional water molecules were observed around the local structural change.

c: In E282A chain B, two additional water molecules were observed in the leftover space upon the exchange. The one proximal to the H-cluster owns a higher B factor.

Supplementary Table 9 | Specific role of each position in the PT-pathway for the proton transfer process according to the conclusions drawn from the present mutagenesis study.

position	functionality
**C299**	Its rather basic thiol-group (*p*Ka:14) mediates proton-transfer between the azadithiolate ligand and the water-cluster (Wat826+Wat1120 in 4XDC). Another water molecule is apparently incapable to rescue the proton transfer function of a missing thiol or aspartic acid residue here. Due to the proximity to the H-cluster, substitutions (Ala, Asp, Ser) decrease the long-term stability of the active site.
**Wat826/ Wat1120**	While not directly examined here, the water-cluster might fulfill a buffer-function which balances p*K*a-difference between C299 and E279 and might further stall the proton transfer function depending on its protonation state.
**E279**	Due to its specific protein environment, this glutamate residue exhibits an increased *pK*a in the neutral to slightly basic range (8). It is therefore quite flexibly protonated and deprotonated which might be required at this median position as it is neither directly influenced by the redox state of the active site, nor by the pH of the bulk solution. When substituting E279, proton transfer function is only partially rescued in case of a conservative exchange to Asp, demonstrating that the carboxy-group is vital here.
**S319**	Its 'permanently' protonated hydroxyl-group very likely mediates proton transfer between E282 and E279 via deep proton tunneling[12].
**E282**	Entry position for the proton transfer which is surface exposed and thus exhibits the naturally low p*K*a of 3-4. Due to its interactions with surface water molecules its protonation state is immediately influenced by the buffer pH and further fine-tuned by neighboring residues such as the arginyl-group of R286 and nearby histidyl-residues. If non-conservatively substituted by a smaller amino acid, the proton transfer function of this surface residue can be partially rescued by invading H_2O molecules.
**R286**	Bystander residue which modulates the *p*Ka of E282 and participates in an H-bond-network that coordinates water molecules at the entrance of the proton transfer pathway.

Supplementary Discussion

The combination of calculated pK_a-values and H-bond distances described above suggests for H_{ox} the H-bond pattern 1 depicted in **Fig. 5** and **Supplementary Fig. 9**. Following the catalytic direction of H_2-oxidation at pH 8, H_2 is heterolytically split at the active center between Fe_d (Lewis acid) and the adt ligand (Lewis base) yielding a putative intermediate-state labeled $H_{hyd}H^+$ ($[4Fe4S]_H^{2+}$-$Fe^{II}Fe^{I}(NH_2^+)[H^-]$)[7](**Supplementary Fig. 9**).

A protonation of the adt-ligand as a consequence of H_2-heterolysis would affect the overall H-bond pattern as it abolishes the interaction between the thiol-group of C299 and adt, while instead strengthening the H-bond between C299 and Wat826. The correspondingly stronger acidity of Wat826 would support the deprotonation of E279 which again strengthens the H-bond contact to S319. This might favor deep proton-tunneling through the ionization resistant hydroxyl-group. The resulting protonation of E_{282} would intermittently interrupt the salt-bridge contact to R286 completing H-bond pattern 2. The H-cluster would be in the hypothetical transient state $H_{Hyd}H^+$ with both H-atoms of the split H_2 still bound to $[2Fe]_H$ (**Supplementary Fig. 9c**). Only an intramolecular electron-transfer from $[2Fe]_H$ to the $[4Fe]_H$ cluster would decrease the basicity of the protonated adt-bridge enough to allow for the proton to be shuttled via the thiol-group to Wat826. The protonated water molecule would support a re-protonation of E279, and at the end of the PT-pathway the release of a proton from E282 to a surface water thus restoring the original state of H-bond contacts (pattern 1) (**Supplementary Fig. 9d**) described for H_{ox}. However, at this point the H-cluster adopted the electron configuration $[4Fe4S]_H^+$-$Fe^{II}Fe^{II}(NH)[H^-]$ which has been verified for H_{hyd} (**Supplementary Fig. 9d**) via EPR spectroscopy by Mulder and coworkers[13]. In the next step, provided that an electron acceptor is available, one of the electrons from the reduced $[4Fe]_H$ cluster is released, initiating a double-oxidation of the H^--intermediate by inducing another intramolecular ET step from $[2Fe]_H$ to $[4Fe]_H$ which restores the reduced $[4Fe]_H$ state and likewise reduces the $[2Fe]_H$-subcluster. An increase in electron density at the $[2Fe]_H$ cluster again strengthens the basicity of the adt-ligand which then accepts the second proton from Fe_d yielding electron configuration $[4Fe4S]_H^+$-Fe(I)Fe(I)(NH_2^+) defined as H_{sred}^*. The protonated adt-ligand again forces the PT-pathway to shift to H-bond pattern 2 (**Supplementary Fig. 9e**). Similar to $H_{hyd}H^+$, H_{sred}^* is a proposed intermediate with a protonated adt-ligand which can be regarded to be a transition-

state rather than a resting state of the catalytic cycle. Upon deprotonation, H-bond pattern 1 is restored again leading to H_{red}'(**Supplementary Fig. 9f**). The recently characterized H_{red}'-state ($[4Fe4S]_H^{1+}$-$Fe^{II}Fe^{I}$(NH)) exhibits the $[2Fe]_H$ redox-configuration of H_{ox} and a reduced $4Fe_H$-cluster similar to H_{hyd} and has been discussed to be one of the main catalytic intermediates instead of the originally proposed single- and double-reduced states H_{red} and H_{sred} [3,6,8,9]. While the geometry and coordination state of H_{red} ($H_{red}H^+$) are still under debate [14] we could demonstrate that both, H_{red} and H_{sred} very likely carry a bridging hydride species (μH^-), which is incompatible with fast catalytic turnover [6,15,] but under steady state conditions both states are assumed to exist in a dynamic equilibrium with H_{red}' and H_{hyd} [3]. Upon oxidizing the reduced $[4Fe]_H$ sub-cluster of H_{red}' the H-cluster returns to the original H_{ox}-state, thus closing the catalytic cycle (**Supplementary Fig. 10b**). Under the conditions of the FTIR-experiment, the last ET-step is substantially inhibited due to the lack of external electron acceptors. When $H_{hyd}H^+$ is regarded as a transition-state, the transition $H_{hyd} \rightarrow H_{red}$' can be viewed as a proton-coupled electron transfer-step (PCET) which relies on a functional PT-pathway. This transition is inhibited in most variants targeting the PT-pathway, demonstrating that this pathway is indeed pivotal for catalytic proton-transfer in terms of substrate/product exchange. Wild type enzyme exhibits an accumulation of H_{hyd} instead of H_{red} and H_{sred} under H_2 only when the buffer pH is significantly decreasing to 4 or under strongly diminished sample humidity. While the competitive transitions from H_{red}' to either H_{sred} or H_{hyd} both involve the uptake of one electron and one proton, it has been shown that at higher pH values the transition to H_{sred} is supported over H_{hyd}[9].

It cannot be ruled out that the residues in the proton transfer pathway adopt alternative configurations in structures of [FeFe]-hydrogenase adjusted to a different redox state. However, no alternative configuration of the PT-pathway has been observed yet, including the crystal structure of 1HFE from DdH, which according to the authors and the shifted μCO ligand represents reduced states (H_{red} or H_{sred}) rather than H_{ox}[16,17].

The validity of this 'bi-stability' model strongly depends on the existence of the yet unidentified states $H_{hyd}H^+$ and H_{sred}^* which unlike the experimentally verified states H_{ox}, H_{hyd} and H_{red}' exhibit a protonated adt bridge. Only the acceptance of states with a protonated adt bridge would require the assignment of a second H-bond pattern. If the adt bridge merely fulfills a

proton shuttle function without the capacity to intermittently bind a proton, this second H-bond pattern can be omitted and a simple 'bucket line' mechanism can be assumed.

Supplementary References:

1 Dörner, E. & Boll, M. Properties of 2-oxoglutarate: ferredoxin oxidoreductase from *Thauera aromatica* and its role in enzymatic reduction of the aromatic ring. *J. Bacteriol.* **184**, 3975-3983 (2002).

2 Lampret, O. *et al.* Interplay between the CN^- ligands and the secondary coordination sphere of the H-cluster in [FeFe]-hydrogenases. *J. Am. Chem. Soc.* **139**, 18222-18230 (2017).

3 Senger, M. *et al.* Protonation/reduction dynamics at the [4Fe–4S] cluster of the hydrogen-forming cofactor in [FeFe]-hydrogenases. *Phys. Chem. Chem. Phys.* **20**, 3128-3140 (2018).

4 Mulder, D. W. *et al.* Stepwise [FeFe]-hydrogenase H-cluster assembly revealed in the structure of $HydA^{\Delta EFG}$. *Nature* **465**, 248-251 (2010).

5 Esselborn, J. *et al.* A structural view of synthetic cofactor integration into [FeFe]-hydrogenases. *Chem. Sci.* **7**, 959-968 (2016).

6 Mebs, S. *et al.* Bridging hydride at reduced H-cluster species in [FeFe]-Hydrogenases revealed by infrared spectroscopy, isotope editing, and quantum Chemistry. *J. Am. Chem. Soc.* **139**, 12157-12160 (2017).

7 Reijerse, E. J. *et al.* Direct observation of an iron-bound terminal hydride in [FeFe]-hydrogenase by nuclear resonance vibrational spectroscopy. *J. Am. Chem. Soc.* **139**, 4306-4309 (2017).

8 Senger, M. *et al.* Proton-coupled reduction of the catalytic [4Fe-4S] cluster in [FeFe]-hydrogenases. *Angew. Chem. Int. Ed.* **56**, 16503-16506 (2017).

9 Sommer, C. *et al.* Proton coupled electronic rearrangement within the H-cluster as an essential step in the catalytic cycle of [FeFe] hydrogenases. *J. Am. Chem. Soc.* **139**, 1440-1443 (2017).

10 Winkler, M. *et al.* Accumulating the hydride state in the catalytic cycle of [FeFe]-hydrogenases. *Nature communications* **8**, 16115 (2017).

11 Mebs, S. *et al.* Hydrogen and oxygen trapping at the H-cluster of [FeFe]-hydrogenase revealed by site-selective spectroscopy and QM/MM calculations. *Biochim. Biophys. Acta* **1859**, 28-41 (2018).

12 Salna, B., Benabbas, A., Sage, J. T., van Thor, J. & Champion, P. M. Wide-dynamic-range kinetic investigations of deep proton tunnelling in proteins. *Nat. Chem.* **8**, 874-880 (2016).

13 Mulder, D. W., Guo, Y., Ratzloff, M. W. & King, P. W. Identification of a catalytic iron-hydride at the H-cluster of [FeFe]-hydrogenase. *J. Am. Chem. Soc.* **139**, 83-86 (2017).

14 Ratzloff, M. W. *et al.* CO-bridged H-cluster intermediates in the catalytic mechanism of [FeFe]-hydrogenase CaI. *J. Am. Chem. Soc.* **140**, 7623-7628 (2018).

15 Chernev, P. *et al.* Hydride binding to the active site of [FeFe]-hydrogenase. *Inorg. Chem.* **53**, 12164-12177 (2014).

16 Nicolet, Y., Piras, C., Legrand, P., Hatchikian, C. E. & Fontecilla-Camps, J. C. *Desulfovibrio desulfuricans* iron hydrogenase: the structure shows unusual coordination to an active site Fe binuclear center. *Structure* **7**, 13-23 (1999).

17 Nicolet, Y. *et al.* Crystallographic and FTIR spectroscopic evidence of changes in Fe coordination upon reduction of the active site of the Fe-only hydrogenase from *Desulfovibrio desulfuricans*. *J. Am. Chem. Soc.* **123**, 1596-1601 (2001).
