# Supplementary material for: Crystallographic and spectroscopic assignment of the proton transfer pathway in [FeFe]-hydrogenases
Source: Nat Commun. 2018 Nov 9;9:4726. doi: 10.1038/s41467-018-07140-x (PMC6226526; doi:10.1038/s41467-018-07140-x)
Supplement: Supplementary file 3 — Reporting Summary [file 41467_2018_7140_MOESM3_ESM.pdf]

naturerresearch

Corresponding author(s): Martin Winkler; Thomas Happe

Reporting Summary

Nature Research wishes to improve the reproducibility of the work that we publish. This form provides structure for consistency and transparency in reporting. For further information on Nature Research policies, see Authors & Referees and the Editorial Policy Checklist.

Statistical parameters

When statistical analyses are reported, confirm that the following items are present in the relevant location (e.g. figure legend, table legend, main text, or Methods section).

n/a	Confirmed	
☐	☒	The exact sample size (*n*) for each experimental group/condition, given as a discrete number and unit of measurement
☐	☒	An indication of whether measurements were taken from distinct samples or whether the same sample was measured repeatedly
☒	☐	The statistical test(s) used AND whether they are one- or two-sided<br>*Only common tests should be described solely by name; describe more complex techniques in the Methods section.*
☒	☐	A description of all covariates tested
☒	☐	A description of any assumptions or corrections, such as tests of normality and adjustment for multiple comparisons
☐	☒	A full description of the statistics including central tendency (e.g. means) or other basic estimates (e.g. regression coefficient) AND variation (e.g. standard deviation) or associated estimates of uncertainty (e.g. confidence intervals)
☒	☐	For null hypothesis testing, the test statistic (e.g. *F*, *t*, *r*) with confidence intervals, effect sizes, degrees of freedom and *P* value noted<br>*Give P values as exact values whenever suitable.*
☒	☐	For Bayesian analysis, information on the choice of priors and Markov chain Monte Carlo settings
☒	☐	For hierarchical and complex designs, identification of the appropriate level for tests and full reporting of outcomes
☒	☐	Estimates of effect sizes (e.g. Cohen's *d*, Pearson's *r*), indicating how they were calculated
☐	☒	Clearly defined error bars<br>*State explicitly what error bars represent (e.g. SD, SE, CI)*

Our web collection on statistics for biologists may be useful.

Software and code

Policy information about availability of computer code

Data collection	GC Solution (Shimadzu 2010) for H2 production data collection; OPUS 7.5(Bruke) for IR data collection; MXCuBe (version 2) and DA+ (only one version) for crystal data collection in ESRF and SLS respectively.
Data analysis	For structural data processing, we used the following programes: XDS (versions between Nov 3, 2014 and Oct 15, 2015), XSCALE (versions between Nov 3, 2014 and Oct 15, 2015) and PHENIX (1.9-1692). Pymol 1.7 was used to show the structural models and electron densities. IR data and enzyme activity data were analyzed by using Origin 9.0.

For manuscripts utilizing custom algorithms or software that are central to the research but not yet described in published literature, software must be made available to editors/reviewers upon request. We strongly encourage code deposition in a community repository (e.g. GitHub). See the Nature Research guidelines for submitting code & software for further information.

Data

Policy information about availability of data

All manuscripts must include a data availability statement. This statement should provide the following information, where applicable:

- Accession codes, unique identifiers, or web links for publicly available datasets
- A list of figures that have associated raw data
- A description of any restrictions on data availability

The coordinates and structure factors for all structures are deposited in the PDB as 6GLY [https://www.rcsb.org/structure/6GLY], 6GLZ [https://www.rcsb.org/structure/6GLZ], 6GM0 [https://www.rcsb.org/structure/6GM0], 6GM1 [https://www.rcsb.org/structure/6GM1], 6GM2 [https://www.rcsb.org/structure/6GM2], 6GM3 [https://www.rcsb.org/structure/6GM3], 6GM4 [https://www.rcsb.org/structure/6GM4], 6GM5 [https://www.rcsb.org/structure/6GM5], 6GM6 [https://www.rcsb.org/structure/6GM6], 6GM7 [https://www.rcsb.org/structure/6GM7] and 6GM8 [https://www.rcsb.org/structure/6GM8]. Further data supporting findings of this study are available from the corresponding authors upon reasonable request. The source data underlying Figs 1b, 2 and Supplementary Figs 1, 2 and 3 are provided as a Source Data file.

Field-specific reporting

Please select the best fit for your research. If you are not sure, read the appropriate sections before making your selection.

☒ Life sciences ☐ Behavioural & social sciences ☐ Ecological, evolutionary & environmental sciences

For a reference copy of the document with all sections, see nature.com/authors/policies/ReportingSummary-flat.pdf

Life sciences study design

All studies must disclose on these points even when the disclosure is negative.

Sample size	As sample size (number of samples measured for each data point) we chose 3 x 3= 9. We collected the data from at least three independent measurements with each three replicates per test series. With this sample size we routinely gain high reproducibility values (low standard deviations) with wild type enzyme.
Data exclusions	No data were excluded
Replication	To verify reproducibility we tested individual enzyme variants in triplicates in a standardized procedure under exactly the same conditions together with wild type enzyme in the same test series and repeated these measurements at least three times. The limited standard deviation documents the high reproducibility of our measurements.
Randomization	Randomization wasn't relevant or necessary here, we tested mutagenized enzyme variants on specific enzymatic features in comparison to the respective wild type enzyme to identify structure function relationships. We always tested (one by one) single variants (in at least triplicates) together in the same test series with the respective wild type enzyme at e.g. defined pH values in a standardized test assay. To randomize here would be too confusing and not helpful.
Blinding	Blinding wasn't necessary here (see answer to Randomization)

Reporting for specific materials, systems and methods

Materials & experimental systems

n/a	Involved in the study
☒	☐ Unique biological materials
☒	☐ Antibodies
☒	☐ Eukaryotic cell lines
☒	☐ Palaeontology
☒	☐ Animals and other organisms
☒	☐ Human research participants

Methods

n/a	Involved in the study
☒	☐ ChIP-seq
☒	☐ Flow cytometry
☒	☐ MRI-based neuroimaging
